# SpyGlass DS-Directed Radiofrequency Ablation With Double Biliary Metal Stent Placement for Managing Recurrent Obstructive Jaundice Secondary to Castleman Disease: A Case Report of a Rare Disease

**DOI:** 10.3389/fsurg.2022.800050

**Published:** 2022-03-02

**Authors:** Yongjin Chen, Chang Fu, Junhong Chen, Weicong Pan, Yu Fu, Kai Liu

**Affiliations:** Department of Hepatopancreatobiliary Surgery, The First Hospital of Jilin University, Changchun, China

**Keywords:** Castleman disease, radiofrequency ablation, cholangioscopy, SpyGlass DS, biliary tract, obstructive jaundice

## Abstract

Castleman disease (CD) rarely presents with obstructive jaundice, which poses a diagnostic and therapeutic challenge to the management of the disease. A 40-year-old man was referred to our hospital for emergent management of upper abdominal pain. An abdominal mass was removed, and the postoperative pathology showed retroperitoneum CD, which was subsequently managed by adjuvant therapy of combination chemotherapy and steroids. One month later, a biliary metal stent was placed due to the presentation of obstructive jaundice. After ~3 months, the patient experienced another episode of obstructive jaundice, and SpyGlass DS cholangioscopy was performed *via* the biliary tract for biopsy, which pathologically showed biliary malignancies. Radiofrequency ablation was performed with a probe, and another uncovered metal stent was placed within the existing metal stent. No stent occlusion occurred during a 6-month follow-up period. In conclusion, CD rarely presents with obstructive jaundice, and a combination of radiofrequency ablation with metal stent implantation under cholangioscopy can prolong the stent patency time and the survival time of patients.

## Introduction

Castleman disease (CD) is a rare lymphoproliferative disorder that was first described by Benjamin Castleman (1). Clinically, CD can be divided into unicentric and multicentric subtypes, which lead to different prognoses (2). Radical surgical resection with adjuvant chemoradiotherapy is the treatment of choice. In cases of multiple metastases, palliative care may be the only option. CD rarely presents with obstructive jaundice. At present, only 12 relevant case reports can be found in the literature, and most of the patients in these reports received radical operations (3). Radiofrequency ablation (RFA) has been proven to be a safe and effective method for the treatment of biliary malignancies (4). In 2015, the novel digital single-operator cholangioscopy system (SpyGlass DS) was introduced and provides high-resolution images for visualization of the biliary tract and biopsy biliary stricture for establishing a definitive diagnosis (5). Here, we report the first case of multicentric CD (MCD) that involved the biliary tract and presented with obstructive jaundice, which was further treated by RFA and metal stent placement under SpyGlass DS cholangioscopy and finally achieved complete and durable remission.

## Case Description

**Figure 1:**
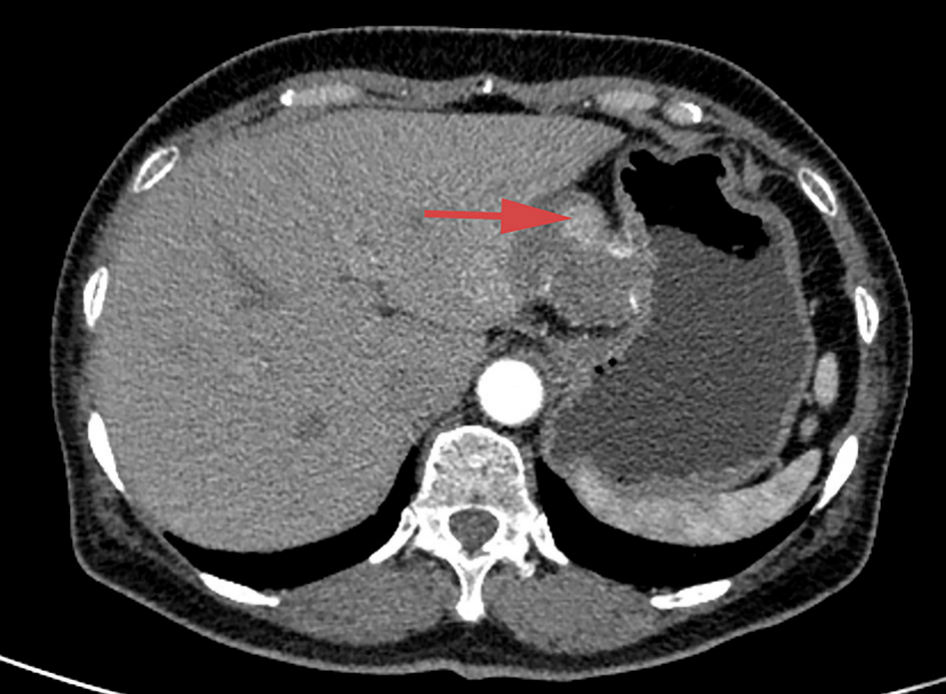
Space occupying lesion, measuring about 6 × 5 cm, in the patient's abdomen.

**Figure 2:**
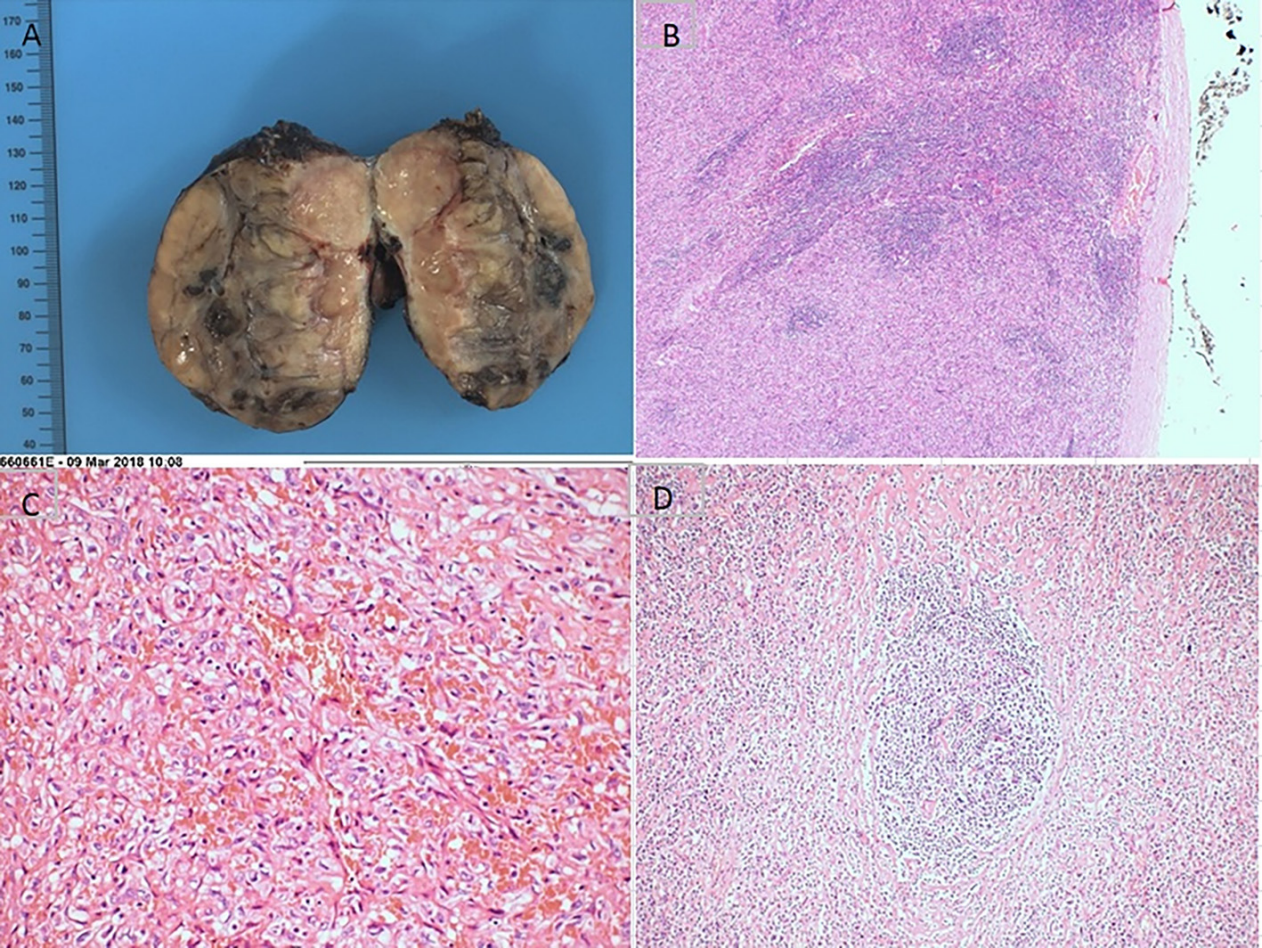
Pathohistological features of mixed type Castleman disease. **(A)** The cutting surface; **(B)** the intact tumor envelope; **(C)** infiltrating neoplastic cells with marked pleomorphism; **(D)** rod-shaped glassy changes were seen in the germinal center.

**Figure 3:**
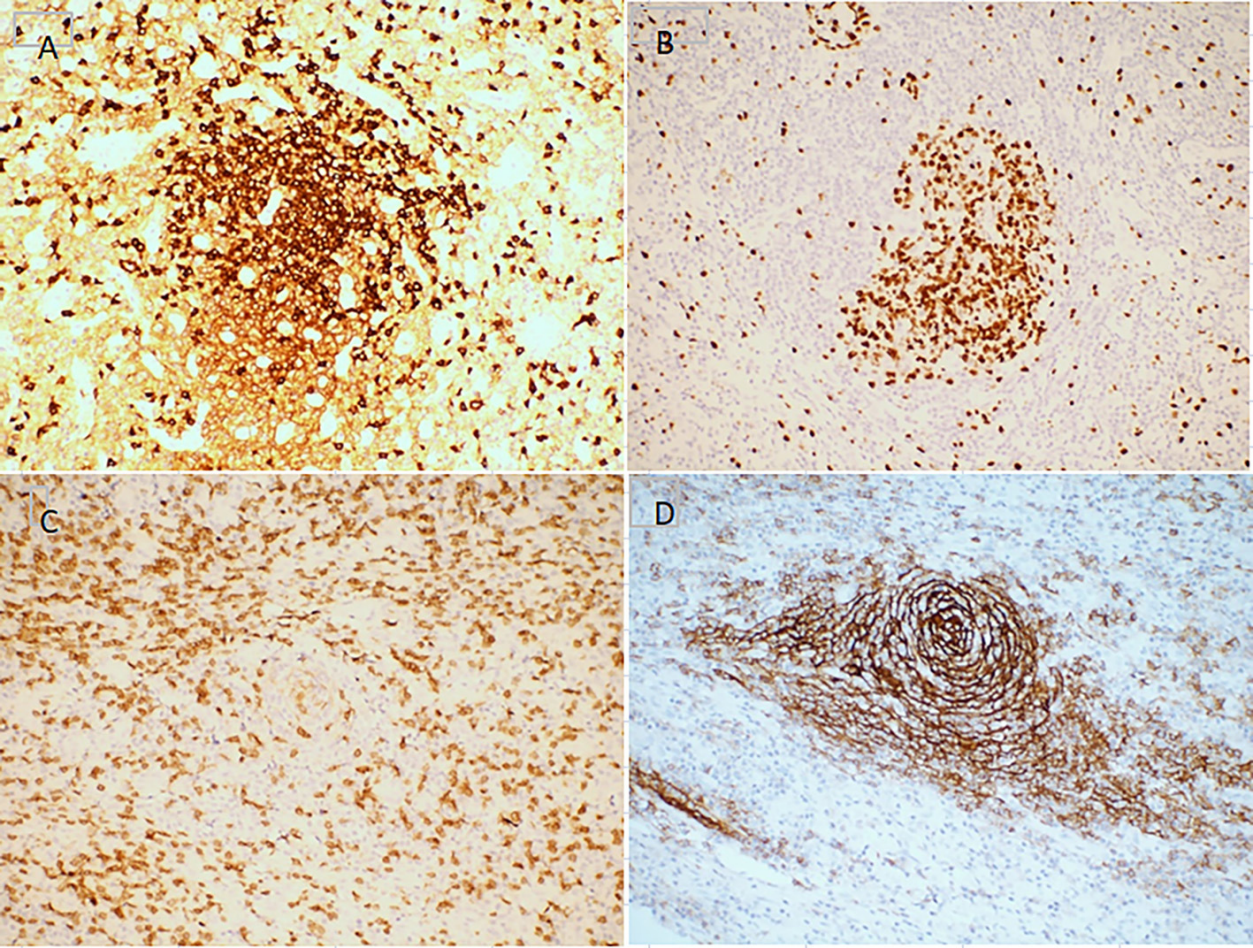
Immunohistochemistry features of mixed type Castleman disease. Hematoxylin staining. Magnification, ×200. **(A)** CD3; **(B)** CD20; **(C)** CD21; **(D)** Ki-67.

The study was approved by the institutional research ethics committee of the First Hospital of Jilin University, under the project identification code 19K041-001, June 2019. Written informed consent was obtained from each subject. A 40-year-old man presented to our hospital with ictericia, sclera, and dark urine lasting for 5 days. The patient had undergone celiac tumor resection in our hospital 4 months previously with a postoperative pathological diagnosis of retroperitoneum CD with positive hilar and periportal lymph nodes (Figures 1–3). Adjuvant therapy of combination chemotherapy and steroids had been administered. Moreover, the patient underwent endoscopic retrograde cholangiopancreatography (ERCP) with metal stent placement 1 month after the initial resection for unknown origin obstructive jaundice. Examination after admission revealed a total bilirubin concentration of 180.7 μmol/L (normal range <26 μmol), direct bilirubin concentration of 157.3 μmol/L (normal range <26 μmol), CA19-9 concentration of 72.3 U/ml, CA-125 concentration of 21.6 U/ml, alpha-fetoprotein concentration of 2.37 ng/ml, carcinoembryonic antigen concentration of 5.59 ng/ml, and C-reactive protein concentration of 21.3 mg/L.

**Figure 4:**
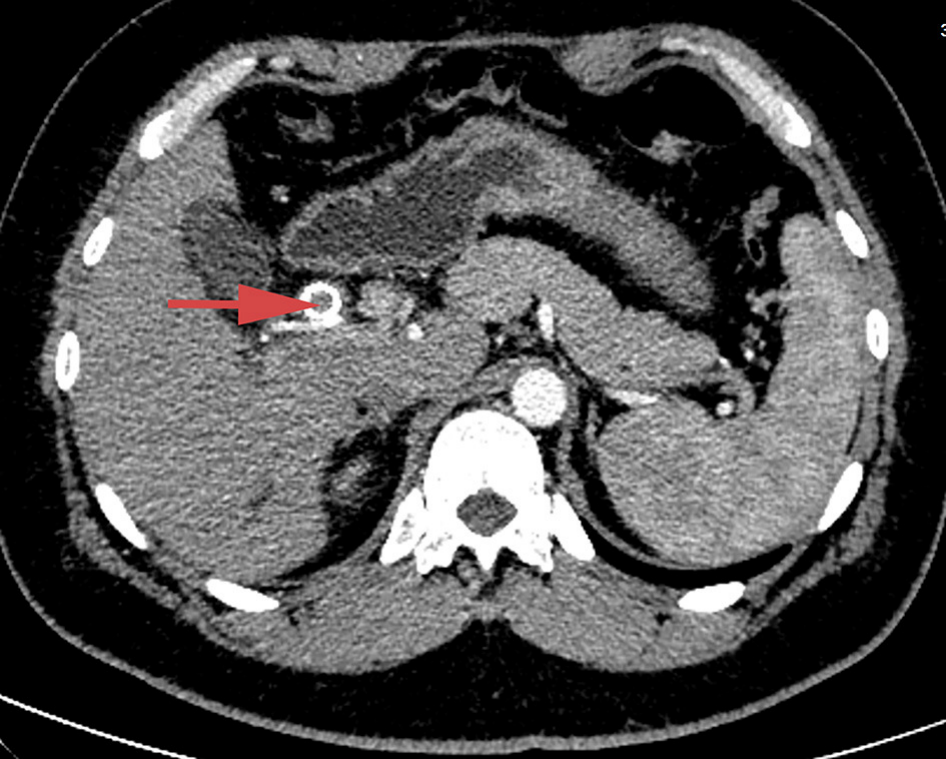
Abdominal contrast-enhanced CT scan, showing high-density shadow in the initially placed biliary metal stent and lumen obstruction.

**Figure 5:**
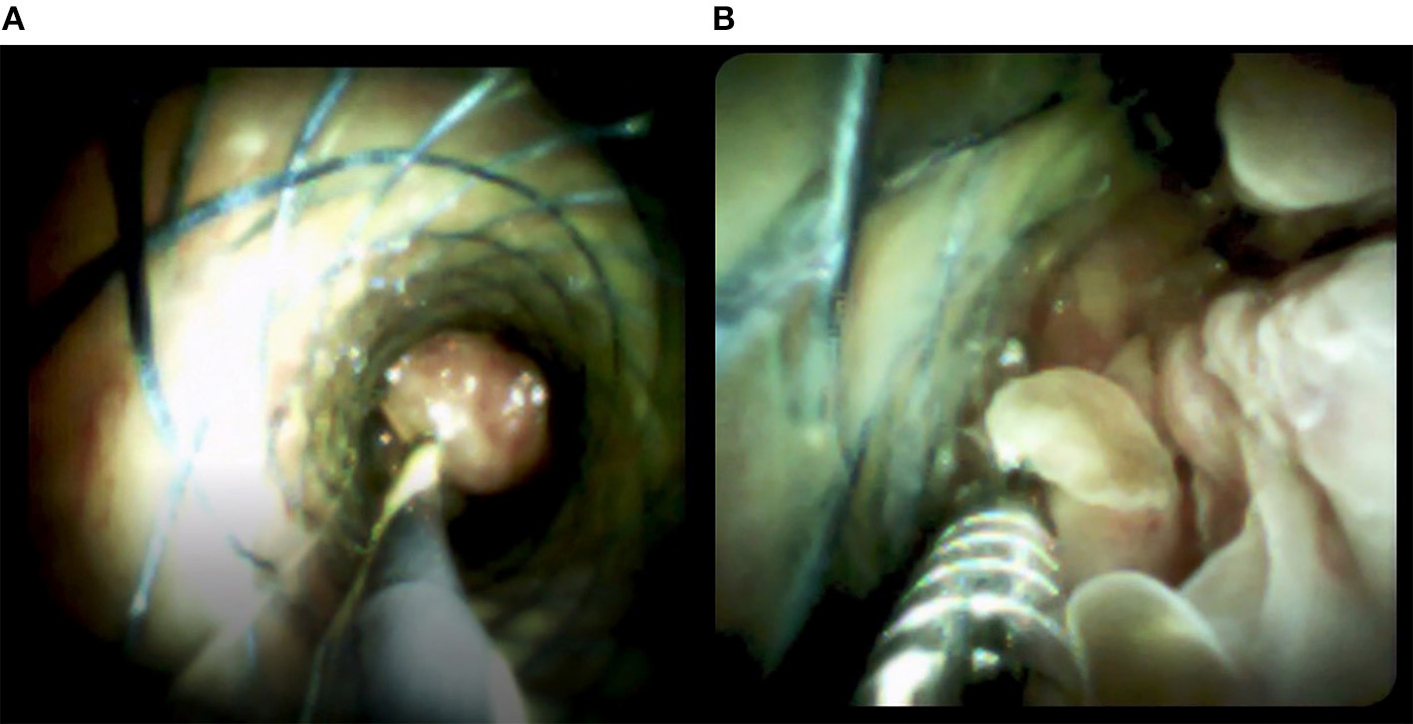
Images from the SpyGlass DS system show **(A)** multiple tumor-like areas of hyperplasia in the bile cavity and **(B)** biopsies taken by SpyBite.

**Figure 6:**
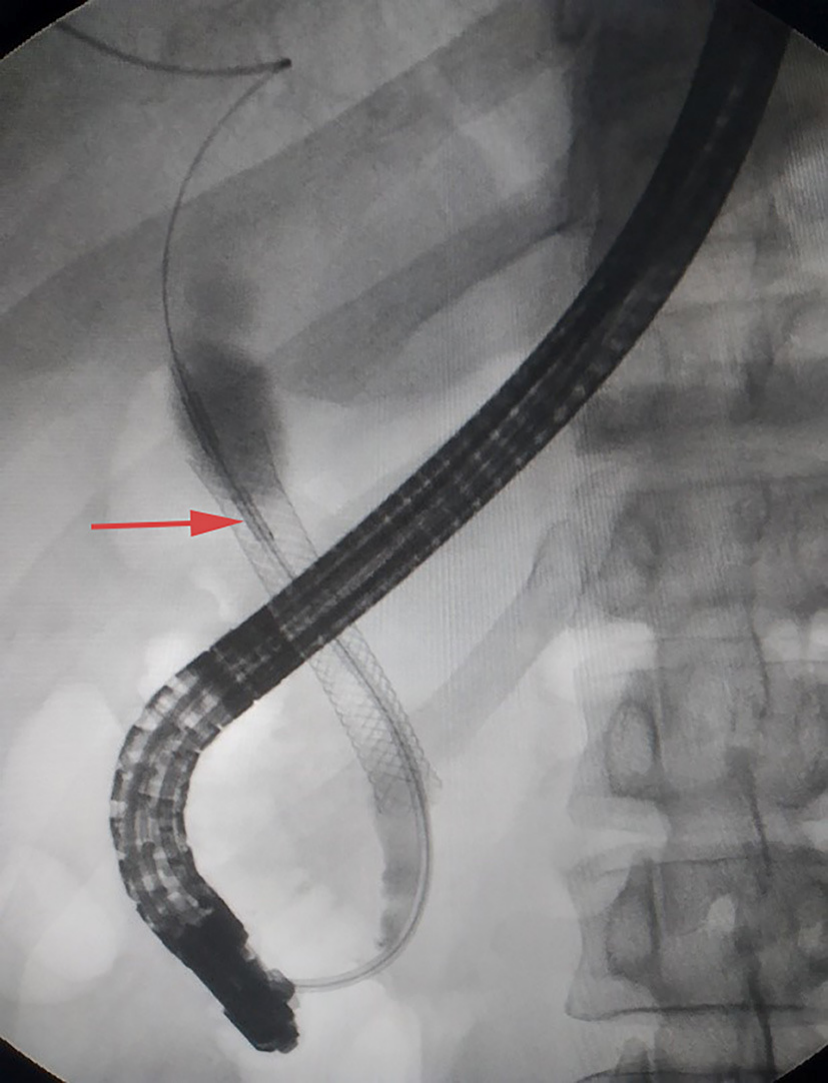
Radiofrequency ablation in the same plane with SpyGlass DS.

**Figure 7:**
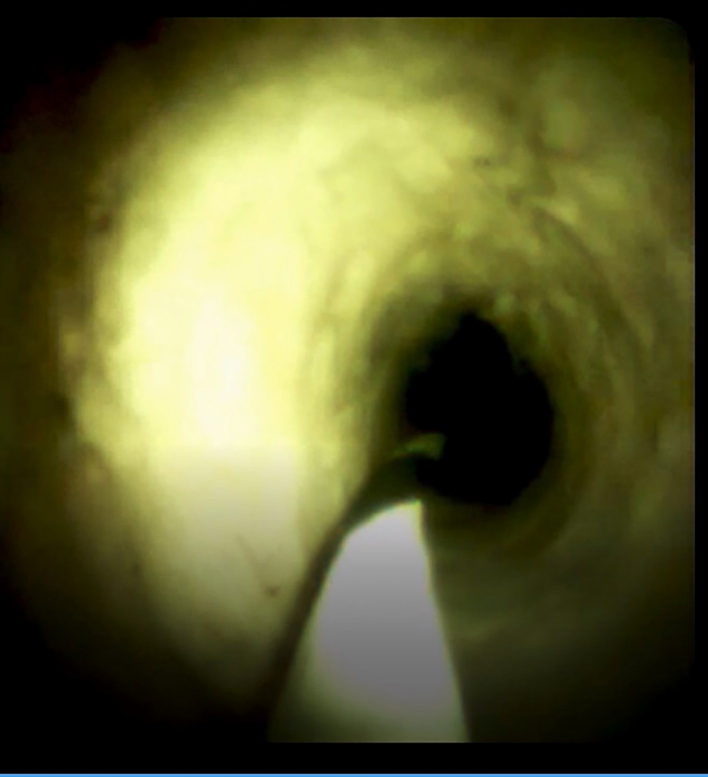
SpyGlass DS system image showing the change after radiofrequency ablation.

**Figure 8:**
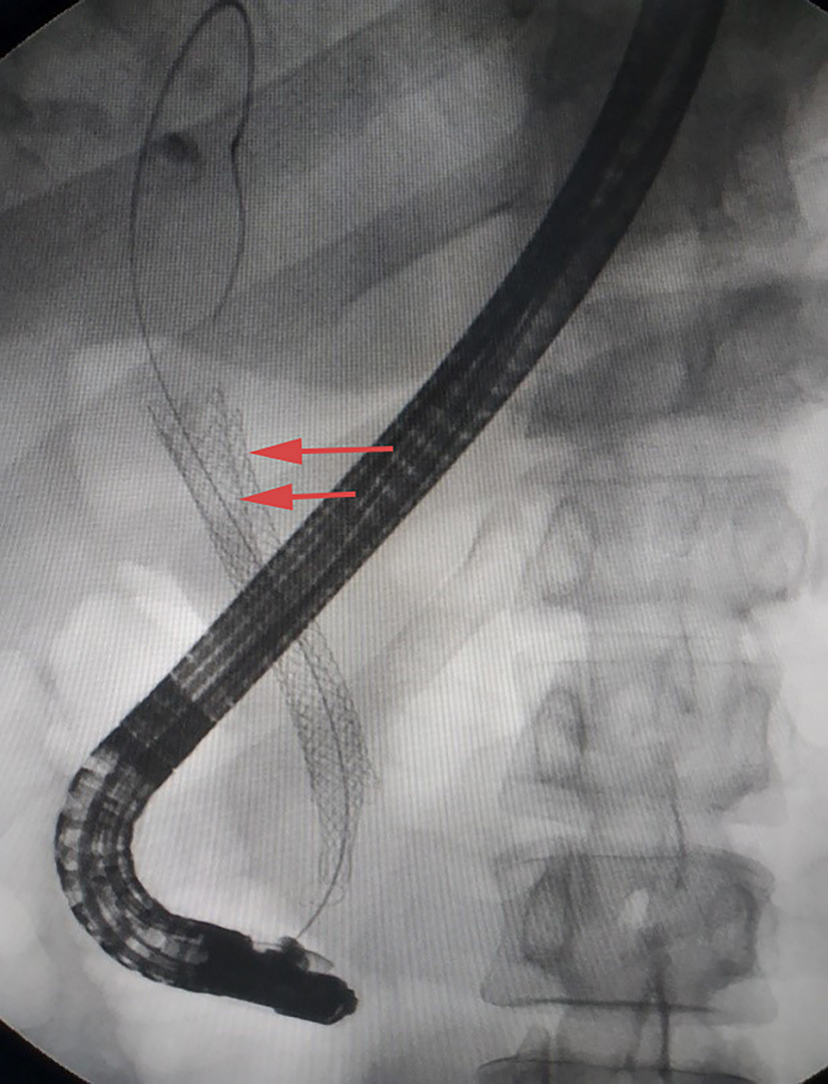
X-ray image showing double stent after insertion of the second biliary uncovered metal stent (yellow arrow).

Enhanced computed tomography (CT) scanning revealed a high-density shadow in the initially placed biliary metal stent, suggesting lumen obstruction (Figure 4). The patient had undergone celiac tumor resection in our hospital 4 months previously with possible severe abdominal adhesions. The patient's general condition was poor, with significant jaundice and severely impaired liver function, making surgery a high-risk option. The patient and his family refused to undergo another open surgery. ERCP was repeated, and multiple tumor-like areas of hyperplasia could be seen in the lumen of the metal stent, and SpyBite biopsies were collected *via* SpyGlass DS (Boston Scientific, Natick, Mass, USA) (Figure 5). The final diagnosis was CD with bile duct metastasis. RFA is a heat-based local ablative therapy that causes coagulative necrosis and tumor destruction. To reduce tumor load and prolong SEMS patency, the endobiliary radiofrequency ablation (ERFA) technique was developed, which has direct necrotic effects on local tumors. It has been successfully used in the endoscopic treatment of malignant biliary obstruction. RFA (10 W, 2 min) was performed (Figure 6). SpyGlass DS imaging showed changes after RFA (Figure 7). A second biliary uncovered metal stent was inserted, and X-ray imaging showed the double stent (Figure 8). Finally, no occlusion of the stent occurred during 6 months of follow-up.

## Discussion

CD is a rare disease entity in which the causes of reactive lymphadenopathy are unknown. Pathologically, CD features obvious proliferation of lymphoid follicles, blood vessels as well as plasma cells to varying degrees (2), and further can be divided into the hyaline-vascular type and plasma cell type. Clinically, it is characterized by significant enlargement of deep or superficial lymph nodes and, in some patients, systemic symptoms and/or multiple system damage.

The disease is classified as unicentric CD (UCD) or MCD subtypes. UCD is more common in young people; pathologically, 90% of UCD cases are the hyaline-vascular type, and surgical resection of enlarged lymph nodes offers a good prognosis. In comparison, MCD is less common than UCD, and patients with MCD always present with enlargement of multiple lymph nodes in combination with systemic symptoms. In some cases, these symptoms include only fever or liver splenomegaly. However, in 20–30% of patients, MCD manifests as nephrotic syndrome, amyloidosis, myasthenia gravis, Kaposi's sarcoma, or B-cell lymphoma. MCD is usually invasive and associated with infection, and the prognosis is poor when it is associated with malignant transformation or lymphoma (6).

To date, 13 cases of CD with jaundice, including the present case, have been reported in the English literature (3), including 7 MCD and 6 UCD cases. Histopathologically, seven cases were diagnosed as hyaline vascular type, three cases as plasma cell type, and three cases as the mixed type. In all UCD cases, resection was successful, and symptoms disappeared completely during follow-up. In contrast, cases of MCD were partially controlled or relapsed.

The involvement of the biliary tract poses diagnostic and therapeutic challenges in the management of CD. A systematic review of nine studies with 505 patients (7) found that RFA combined with biliary stent placement offered a pooled mean difference of 50.6 days in stent patency (95% CI, 32.83–68.48) and had a longer overall survival (hazard ratio, 1.395; 95% CI, 1.145–1.7; *P* < 0.001) in comparison with stent placement alone. Thus, the efficacy and safety of RFA for managing unresectable extrahepatic cholangiocarcinoma have been demonstrated.

SpyGlass DS with direct visualization and biopsy for diagnosis of biliary tract diseases has been shown to be safe and to offer a high success rate (8). RFA for malignant biliary stricture using SpyGlass DS not only retains the safety but also increases the success rate of the treatment. In addition, SpyBite can easily execute biopsies under SpyGlass DS, leading to improved diagnostic accuracy (9–11).

In summary, we reported the first case of multicentric CD presenting with jaundice due to biliary tract re-occlusion, which was treated by SpyGlass DS-directed RFA and double metal stent insertion, subsequently leading to complete and durable remission of the disease.

## Conclusion

CD rarely presents as jaundice due to obstruction of the biliary tract. However, such cases can be feasibly and effectively managed by SpyGlass DS-directed RFA with metal stent insertion, which may improve stent patency time and survival time.

## Data Availability Statement

The original contributions presented in the study are included in the article/supplementary material, further inquiries can be directed to the corresponding author/s.

## Ethics Statement

Ethical review and approval was not required for the study on human participants in accordance with the local legislation and institutional requirements. Written informed consent for participation was not required for this study in accordance with the national legislation and the institutional requirements.

## Author Contributions

KL was involved in the patient's treatment. YC and CF prepared and edited the manuscript. JC and WP gathered detailed clinical information. YF conducted the pathological examination and took pictures. All authors contributed to the article and approved the submitted version.

## Funding

Supported by the Jilin Science and Technology Development Program (CN) (No. 20191102031YY).

## Conflict of Interest

The authors declare that the research was conducted in the absence of any commercial or financial relationships that could be construed as a potential conflict of interest.

## Publisher's Note

All claims expressed in this article are solely those of the authors and do not necessarily represent those of their affiliated organizations, or those of the publisher, the editors and the reviewers. Any product that may be evaluated in this article, or claim that may be made by its manufacturer, is not guaranteed or endorsed by the publisher.
